# Interplay between sirtuins, MYC and hypoxia-inducible factor in cancer-associated metabolic reprogramming

**DOI:** 10.1242/dmm.016287

**Published:** 2014-08-01

**Authors:** Bernadette M. M. Zwaans, David B. Lombard

**Affiliations:** Department of Pathology and Institute of Gerontology, University of Michigan, Ann Arbor, MI 48109, USA

**Keywords:** Sirtuins, MYC, HIF, Warburg effect, Metabolic reprogramming

## Abstract

In the early twentieth century, Otto Heinrich Warburg described an elevated rate of glycolysis occurring in cancer cells, even in the presence of atmospheric oxygen (the Warburg effect). Despite the inefficiency of ATP generation through glycolysis, the breakdown of glucose into lactate provides cancer cells with a number of advantages, including the ability to withstand fluctuations in oxygen levels, and the production of intermediates that serve as building blocks to support rapid proliferation. Recent evidence from many cancer types supports the notion that pervasive metabolic reprogramming in cancer and stromal cells is a crucial feature of neoplastic transformation. Two key transcription factors that play major roles in this metabolic reprogramming are hypoxia inducible factor-1 (HIF1) and MYC. Sirtuin-family deacetylases regulate diverse biological processes, including many aspects of tumor biology. Recently, the sirtuin SIRT6 has been shown to inhibit the transcriptional output of both HIF1 and MYC, and to function as a tumor suppressor. In this Review, we highlight the importance of HIF1 and MYC in regulating tumor metabolism and their regulation by sirtuins, with a main focus on SIRT6.

## SIRT6: a multi-functional enzyme implicated in diverse pathologies

The sirtuins are a conserved family of NAD^+^-dependent enzymes that regulate many cellular functions, in particular those related to stress responses. The *SIR2* (silent information regulator 2) gene in budding yeast was the first sirtuin to be identified and functionally characterized. The initial interest in sirtuins was related to their roles in regulating lifespan. Several groups reported that an increased dosage of *SIR2* and its homologs promotes increased lifespan in budding yeast, worms and flies (Hoffmann et al., 2013; Mouchiroud et al., 2013; Stumpferl et al., 2012; Viswanathan and Guarente, 2011). As an aside, it is should be noted that one prominent study did not reproduce these pro-longevity effects of sirtuins in worms or flies (Burnett et al., 2011), and aspects of the roles of sirtuins in yeast longevity are also still hotly debated (Longo and Kennedy, 2006). The discrepant results obtained by different laboratories likely result from variations in experimental protocols, strain backgrounds and/or husbandry conditions. Notwithstanding these controversies, the apparently conserved pro-longevity effects of invertebrate sirtuins have led to intensive efforts to characterize the functions of the seven mammalian sirtuins, termed SIRT1-SIRT7.

Each of the mammalian sirtuins possesses a conserved catalytic domain; however, sirtuins differ at their N- and C-termini, and are a divergent family in terms of subcellular localization, targets and functions (Giblin et al., 2014). Biochemically, mammalian sirtuins primarily act as NAD^+^-dependent lysine deacetylases, with varied catalytic efficiencies and substrates. Two mammalian sirtuins, SIRT4 and SIRT6, have been shown to possess biologically relevant mono-ADP-ribosyltransferase activity (Ahuja et al., 2007; Frye, 1999; Haigis et al., 2006; Liszt et al., 2005). Some sirtuins remove non-canonical lysine post-translational modifications such as succinyl, malonyl, glutaryl and acyl groups (Du et al., 2011; Feldman et al., 2013; Jiang et al., 2013; Peng et al., 2011; Tan et al., 2014). Through these activities, mammalian sirtuins modulate a diverse array of biological processes, such as transcriptional regulation, metabolism, genomic stability, cell cycle control and inflammation (Morris, 2013).

In agreement with the results from invertebrate models, it is now known that at least two mammalian sirtuins, SIRT1 and SIRT6, extend lifespan in mice when overexpressed. In the case of SIRT1, brain-specific, but not whole-body, overexpression increases lifespan through increased neural activity in hypothalamic nuclei, leading to improved maintenance of skeletal muscle mitochondrial function and other beneficial effects (Herranz et al., 2010; Satoh et al., 2013). Whole-body SIRT6 overexpression extends lifespan in male mice specifically by attenuating insulin–IGF-1-like signaling (IIS), and potentially by decreasing the incidence of lung carcinoma (Kanfi et al., 2012; Lombard and Miller, 2012).

In line with the putative role of sirtuins in promoting longevity, a large body of evidence has revealed major roles for individual sirtuins in suppressing diverse age-associated pathologies, including cancer and metabolic diseases (Giblin et al., 2014). The primary focus of this Review is the sirtuin SIRT6, and specifically its roles in neoplastic transformation, an area in which there has been a large amount of recent progress, as discussed in depth in the following sections. Molecularly, a major function of SIRT6 is to deacetylate histone H3 acK9 and acK56 (Kawahara et al., 2009; Michishita et al., 2008; Michishita et al., 2009; Yang et al., 2009). In addition to its lysine deacetylase activity, SIRT6 can act as a mono-ADP ribosyltransferase towards PARP-1, and a TNFα deacylase (Jiang et al., 2013; Pan et al., 2011). Binding of free fatty acids to SIRT6 enhances its deacetylase activity *in vitro* (Feldman et al., 2013), although the *in vivo* significance of this finding remains to be established. Through its H3 deacetylase activity, SIRT6 attenuates the transcriptional activity of numerous transcription factors – NF-κB, HIF1, MYC (c-MYC) and JUN – to inhibit target gene expression (Kawahara et al., 2009; Sebastián et al., 2012; Sundaresan et al., 2012; Xiao et al., 2012; Zhong et al., 2010). Likewise, SIRT6 suppresses the activities of the lipogenic transcription factors SREBP1 and SREBP2 through multiple mechanisms (Elhanati et al., 2013; Tao et al., 2013).

SIRT6 has a central role in many processes that are essential for cellular and organismal homeostasis. By attenuating HIF1 transcriptional output and decreasing intracellular insulin signaling, SIRT6 promotes mitochondrial oxidative metabolism and decreases cellular glucose uptake (Xiao et al., 2010; Zhong et al., 2010). In the absence of SIRT6 function, mice develop lethal hypoglycemia (Mostoslavsky et al., 2006; Xiao et al., 2010). In the liver, SIRT6 indirectly suppresses gluconeogenesis by deacetylating and activating the GCN5 acetyltransferase, which in turn acetylates and inhibits the metabolic regulator PGC1α (Dominy et al., 2012). SIRT6 suppresses obesity, fatty liver, metabolic syndrome (Kanfi et al., 2010; Kim et al., 2010b; Schwer et al., 2010), inflammation (Lappas, 2012; Lee et al., 2013; Xiao et al., 2012), cardiac hypertrophy (Sundaresan et al., 2012; Yu et al., 2013) and cellular senescence (Cardus et al., 2013; Kawahara et al., 2009; Shen et al., 2013; Xie et al., 2012). SIRT6 also promotes genomic stability by enhancing the activities of key proteins involved in DNA repair, including PARP-1, CtIP and DNA-PK (Kaidi et al., 2010; Mao et al., 2012; McCord et al., 2009; Toiber et al., 2013). SIRT6 focally deacetylates acH3K56 around DNA double-strand breaks and recruits the chromatin remodeling factor SNF2H, which in turn promotes the recruitment of other repair factors (Toiber et al., 2013). SIRT6 and its *Caenorhabditis elegans* homolog, SIR-2.4, promote the formation of cytoplasmic stress granules, and resistance to heat stress (Jedrusik-Bode et al., 2013; Simeoni et al., 2013).

### SIRT6 in cancer

**Fig. 1.**
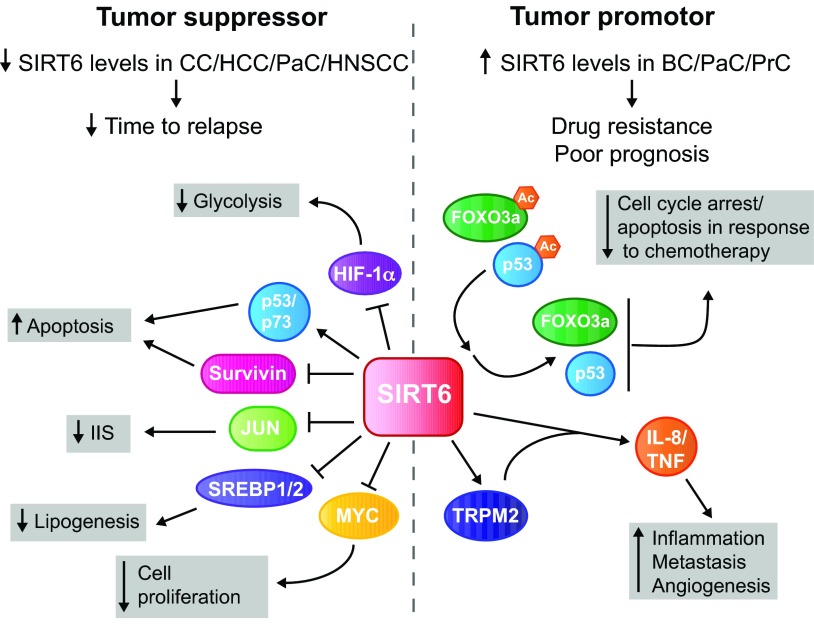
**Dual roles for SIRT6 in cancer.** SIRT6 has been reported to have both tumor suppressor and oncogenic properties. Reduced levels of SIRT6 have been described in colon cancer (CC), hepatocellular carcinoma (HCC), pancreatic cancer (PaC) and head and neck squamous cell carcinoma (HNSCC), correlating with increased cancer stage and grade, and/or with a shortened time to relapse in comparison to tumors with higher levels of SIRT6. SIRT6 can protect against tumorigenesis through multiple pathways: (1) inhibition of HIF-1α and MYC transcriptional activity, which decreases glycolysis and cellular proliferation, respectively; (2) inhibition of the anti-apoptotic factor survivin; (3) activation of the p53 and p73 apoptosis pathways in cancer cells specifically. Furthermore, SIRT6 represses SREBP1 and SREBP2 (SREBP1/2) and JUN activity, resulting in reduced lipogenesis and insulin–IGF-1-like signaling (IIS), respectively. Both of these processes likely impact cancer cell proliferation. By contrast, high SIRT6 levels have been reported in breast cancer (BC), PaC and prostate cancer (PrC), and are associated with drug resistance and poor prognosis. High SIRT6 levels promote cellular proliferation through deacetylation of the cell cycle control proteins FOXO3a and p53, and increase IL-8- and TNF-mediated inflammatory responses, angiogenesis and tumor metastasis in part through activation of the Ca^2+^ channel TRPM2. Ac, acetyl group; TNF, tumor necrosis factor; IL-8, interleukin-8.

The finding that SIRT6 functions in regulating metabolism, genomic stability and cellular senescence – all phenomena relevant for neoplasia – has prompted multiple groups to assess roles for SIRT6 in cancer. Collectively, these studies have shown that SIRT6 plays both oncogenic and tumor suppressor roles, in a cell- and context-specific manner (Fig. 1). Increased SIRT6 expression has been reported in pancreatic, prostate and breast cancers, where high SIRT6 levels are associated with chemotherapy resistance and poor prognosis (Bauer et al., 2012; Khongkow et al., 2013; Liu et al., 2013b). In this context, high levels of SIRT6 promote deacetylation and inactivation of the tumor suppressor proteins FOXO3a and p53, and, in addition, activate production of the pro-inflammatory cytokines TNF and IL-8, in part through the Ca^2+^ channel TRPM2 (Bauer et al., 2012).

Conversely, SIRT6 expression is decreased in human colon, pancreatic, liver, and head and neck squamous cell carcinomas (Lai et al., 2013; Marquardt et al., 2013; Min et al., 2012; Sebastián et al., 2012). Mechanistically, low levels of SIRT6 increase transcriptional output of MYC and HIF1, resulting in accelerated cellular proliferation and glycolysis, respectively (Sebastián et al., 2012), discussed in detail subsequently. Furthermore, decreased SIRT6 levels are associated with increased expression of survivin (an inhibitor of apoptosis), impaired p53 and p73 apoptotic activity, chemotherapy resistance, and metabolic reprogramming (Lai et al., 2013; Marquardt et al., 2013; Min et al., 2012; Sebastián et al., 2012). As mentioned previously, SIRT6 transgenesis extends lifespan in male mice (Kanfi et al., 2012); this effect might occur in part through a reduction in lung cancer incidence in SIRT6 overexpressors. Enterocyte-specific ablation of *Sirt6* exacerbates intestinal tumorigenesis in *Apc^+/min^* mice by inducing a metabolic switch similar to that observed in some cancer cells (Sebastián et al., 2012). This metabolic switch represents a phenomenon originally identified in the context of the Warburg effect.

### Warburg effect

Cells regulate glucose metabolism based on their differentiation status and growth state, and the availability of oxygen. Glycolysis is the metabolic process in which glucose is converted into pyruvate. In differentiated tissues, when oxygen is present, pyruvate then enters the mitochondrial tricarboxylic acid (TCA) cycle to be fully oxidized to CO_2_ (oxidative phosphorylation). However, under hypoxic conditions, pyruvate is instead converted into lactate in anaerobic glycolysis (Vander Heiden et al., 2009).

In 1924, Otto Warburg observed that cancer cells preferentially convert glucose into lactate, irrespective of the presence of oxygen (aerobic glycolysis) (Warburg, 1956). This phenomenon, termed the Warburg effect, is also observed in non-neoplastic proliferating cells, such as lymphocytes (Wu and Zhao, 2013) and lipopolysaccharide (LPS)-stimulated macrophages (Tannahill et al., 2013).

It might at first seem surprising that cancer cells carry out a form of metabolism that is relatively inefficient at generating ATP. For each molecule of glucose that enters the cell, oxidative phosphorylation generates up to roughly 36 molecules of ATP, whereas aerobic glycolysis provides only two net ATP molecules (Vander Heiden et al., 2009). Thus, glycolysis must provide rapidly proliferating cells with benefits that outweigh a lower efficiency of ATP production. A large body of recent work indicates that glycolysis and lactate production provide cancer cells with a number of advantages that drive tumorigenesis. First, in the presence of ample glucose, glycolysis generates ATP more rapidly than oxidative phosphorylation (Wu and Zhao, 2013). Second, aerobic glycolysis provides the cell with an increased capacity to generate precursors for synthesis of macromolecules (lipids, nucleic acids and proteins) that are essential for rapid cell division (Soga, 2013; Vander Heiden et al., 2009; Wu and Zhao, 2013). Finally, lactate secretion by tumors creates a toxic environment for immune cells, thereby inhibiting immune surveillance, and stimulates endothelial cells to form new blood vessels, facilitating tumor metastasis (Hirschhaeuser et al., 2011). Lactate levels are negatively correlated with survival in patients with diverse tumor types, including cervical cancer, head and neck squamous cell carcinoma, and glioblastoma multiforme (Hirschhaeuser et al., 2011). In addition, high blood lactate levels are associated with tumor radioresistance (Hirschhaeuser et al., 2011). It is now known that aerobic glycolysis is a part of the broader metabolic reprogramming that occurs in cancer cells (Ward and Thompson, 2012).

**Fig. 2.**
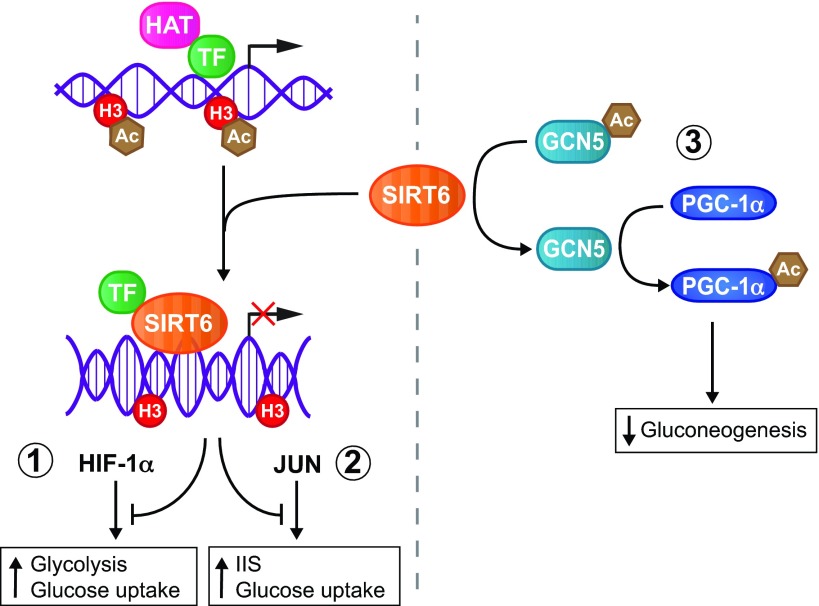
**SIRT6 as a master regulator of glucose metabolism.** SIRT6 regulates glucose metabolism through at least three distinct mechanisms. SIRT6 deacetylates histone H3, thereby attenuating transcriptional output of (1) HIF-1α and (2) JUN, which normally enhance glucose uptake and induce glycolysis or activate the insulin–IGF-1-like signaling (IIS) pathway. (3) SIRT6 deacetylates the histone acetyltransferase GCN5 (KAT2A), which in turn acetylates and activates the transcriptional regulator PPARγ coactivator-1α (PGC-1α), reducing *de novo* production of glucose (gluconeogenesis) in the liver. Ac, acetyl group; HAT, histone acetyltransferase; H3, histone H3; TF, transcription factor.

SIRT6 plays a particularly crucial physiological role in maintaining glucose homeostasis. The first observation linking SIRT6 and glucose metabolism was made in SIRT6-deficient mice. Loss of *Sirt6* in a 129Sv strain background caused severe hypoglycemia that resulted in death by 1 month post-partum (Mostoslavsky et al., 2006). Subsequent studies have shown that this hypoglycemia results from elevated tissue uptake of blood glucose in SIRT6-deficient mice, despite their lower circulating insulin levels. Collectively, these studies have revealed that SIRT6 is a master regulator of glucose homeostasis (Dominy et al., 2012; Kim et al., 2010b; Xiao et al., 2010; Zhong et al., 2010). SIRT6 exerts this function through three distinct mechanisms: (1) inhibiting the activity of HIF-1α, a transcription factor that drives glycolysis and simultaneously inhibits oxidative phosphorylation (Zhong et al., 2010), (2) attenuating IIS and glucose uptake by reducing JUN transcriptional activity (Sundaresan et al., 2012; Xiao et al., 2012), and (3) inhibiting hepatic gluconeogenesis by promoting acetylation of the transcription factor PGC-1α (Dominy et al., 2012) (Fig. 2).

Highlighting the central role of metabolic reprogramming in neoplasia, mutations in genes that encode proteins involved in mitochondrial metabolism can promote tumorigenesis (Wu and Zhao, 2013). A number of transcription factors have been identified as drivers of cancer metabolism (Soga, 2013; Ward and Thompson, 2012). In this Review we will focus on two such transcription factors – HIF-1α and MYC – that have been linked to SIRT6 (Sebastián et al., 2012). Furthermore, we will discuss the roles of other sirtuin family members in modulating HIF-1α and MYC activity. Understanding the mechanistic interplay between sirtuin proteins and these transcription factors might uncover vulnerabilities in cancer cells that could be targeted in the context of novel anti-neoplastic therapies.

## HIF1 is a driver of metabolic reprogramming in cancer

### Hypoxia-inducible factor

The hypoxia-inducible factors (HIFs) – HIF1, HIF2 and HIF3 – are transcription factors that drive glycolysis and lactate production when the oxygen supply is limited. HIFs consist of an α-subunit, levels of which are sensitive to oxygen concentration, and a stable β-subunit. Under physiological oxygen tension, the α-subunit undergoes hydroxylation by prolyl hydroxylase (PHD) and subsequent proteasomal degradation mediated by von Hippel-Lindau (VHL) protein and E3-ligase (Keith et al., 2012; Semenza, 2010). However, hypoxia or mitochondrial reactive oxygen species (ROS) inhibit the activity of PHD, stabilizing the α-subunit of HIF and resulting in the HIF complex binding to hypoxia-responsive elements (HREs) in the promoters of HIF target genes. The ubiquitously expressed HIF-1α was first identified in 1995, followed shortly after by the discovery of HIF-2α (Ema et al., 1997; Flamme et al., 1997; Hogenesch et al., 1997; Tian et al., 1997; Wang et al., 1995). HIF-2α was initially thought to be mainly expressed in endothelial cells; however, expression of HIF-1α and HIF-2α overlaps in many cell types, and they regulate common as well as unique target genes (Hu et al., 2003; Keith et al., 2012; Raval et al., 2005; Wiesener et al., 2003).

HIF1 induces expression of a number of glycolytic genes, such as *SLC2A1* and *SLC2A3*, hexokinases 1 and 2, *LDHA*, *MCT4* and *PDK1*. *SLC2A1* and *SLC2A3* encode the glucose transporters GLUT1 and GLUT3, respectively, which are responsible for basal, non-insulin-responsive glucose uptake. Once imported into the cell, glucose is converted to glucose-6-phosphate in the initial step of glycolysis by hexokinases. Pyruvate generated in the final step of glycolysis can either be converted into acetyl-CoA by pyruvate dehydrogenase (PDC) for further metabolism in the TCA cycle, or be converted into lactate. Under hypoxic conditions, HIF1 upregulates expression of glycolytic enzymes and favors lactate production, while inhibiting pyruvate entry into the TCA cycle. Two of HIF1’s major transcriptional targets are pyruvate dehydrogenase kinase 1 (*PDK1*) and lactate dehydrogenase (*LDH*). PDK1 phosphorylates the E1α subunit of PDC, thereby inhibiting holoenzyme activity. LDH catalyzes conversion of pyruvate into lactate. Once formed, lactate is transported out of the cell by the monocarboxylate transporter MCT4 (Keith et al., 2012; Semenza, 2010). Increased expression of HIF-1α and HIF-2α has been detected in many different cancer types as well as in tumor-associated stromal cells, and, in both cases, high HIF levels are associated with a poor clinical outcome (Bonuccelli et al., 2010; Keith et al., 2012; Pavlides et al., 2010; Semenza, 2010).

The finding that increased HIF expression has been observed in both cancer cells as well their associated stromal cells supports the existence of the ‘reverse Warburg effect’. According to this model, stromal cells rather than the cancer cells are reprogrammed to perform aerobic glycolysis in many tumors. Hence, stromal cells generate lactate, ketone bodies and other energy-rich intermediates that can be taken up by neighboring cancer cells to fuel oxidative phosphorylation and mitochondrial metabolism (Martinez-Outschoorn et al., 2012; Sotgia et al., 2012). Unfortunately, little characterization of the roles for sirtuins in tumor stromal cells has been performed to date. Such studies would entail an examination of the properties of sirtuin-proficient tumor allografts implanted into sirtuin-deficient animals, and/or ablation of sirtuin genes specifically in stromal cells. Although potentially providing important new insights, such studies have not yet been described in the literature. Therefore, we focus our discussion on roles for sirtuins in neoplastic cells themselves.

### Impact of sirtuins on HIF activity

**Fig. 3.**
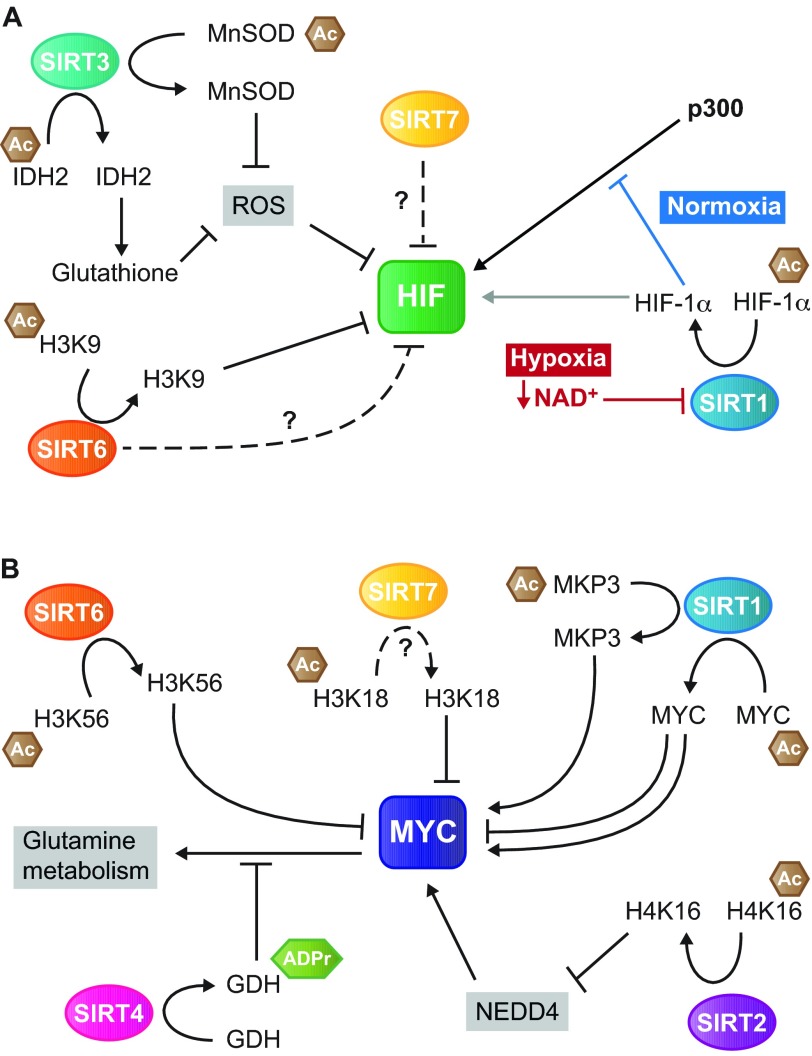
**Roles of sirtuins in regulating MYC- and HIF-mediated tumor metabolic reprogramming.** Schematic overview of the known mechanisms through which the sirtuin proteins regulate the activity of HIF (A) and MYC (B) proteins. (A) SIRT1, SIRT3 and SIRT6 modulate HIF activity through deacetylation of histone (SIRT6) and non-histone (SIRT1/3) proteins. SIRT1 deacetylates HIF-1α directly. Leammle et al. (Leammle et al., 2012) showed that deacetylation of HIF-1α results in elevated transcription of HIF target genes (gray arrow), whereas Lim and colleagues (Lim et al., 2010) found that deacetylated HIF-1α inhibits the recruitment of p300 to the promoters of HIF-1α target genes (blue inhibitory T bar). In addition, SIRT7 also inhibits HIF activity; however, the underlying mechanism is unknown. (B) SIRT1, SIRT2, SIRT4, SIRT6 and SIRT7 all regulate MYC activity, either through deacetylation of histones (SIRT2/6/7) or of other proteins (SIRT1). SIRT4 regulates glutamine metabolism through effects on GDH, downstream of MYC. Ac, acetyl group; ADPr, ADP ribosyl group; MnSOD, manganese superoxide dismutase; IDH2, isocitrate dehydrogenase 2; ROS, reactive oxygen species; HIF, hypoxia inducible factor; NAD^+^, nicotinamide adenine dinucleotide; MKP3, MAP kinase phosphatase 3; GDH, glutamate dehydrogenase; H3K9, histone 3 lysine 9; H3K18, histone 3 lysine 18; H3K56, histone 3 lysine 56; H4K16, histone 4 lysine 16; NEDD4, neural precursor cell expressed developmentally downregulated protein 4.

Sirtuin proteins regulate HIF in a variety of ways: inhibition of transcriptional output (SIRT6), post-translational modification (SIRT1) and regulation of HIF1 stability (SIRT3, SIRT6 and SIRT7) (Fig. 3A and Table 1).

**Table 1.**
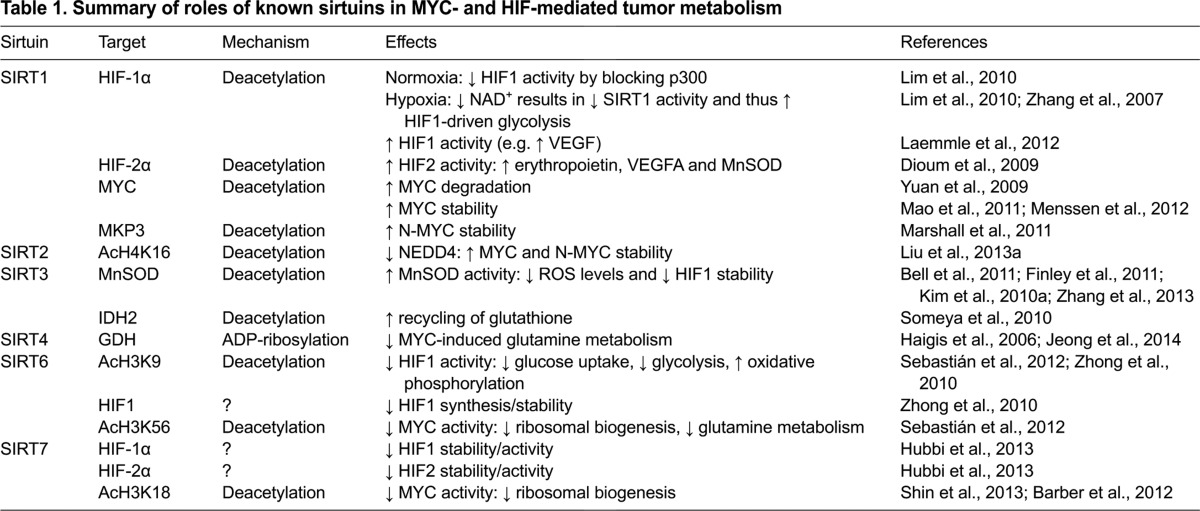
Summary of roles of known sirtuins in MYC- and HIF-mediated tumor metabolism

SIRT6 regulates HIF-1α activity at the chromatin level by deacetylating acH3K9 HIF-1α target genes, thereby attenuating HIF-1α transcriptional output (Zhong et al., 2010). SIRT6 deficiency also enhances HIF-1α protein synthesis and stability, although the mechanistic details of this remain unclear (Zhong et al., 2010). Consequently, *Sirt6* deletion results in elevated HIF-1α levels and activity, driving increased expression of *Glut1*, *Ldha* and *Pdk1* (Zhong et al., 2010). As mentioned earlier, SIRT6 acts as a tumor suppressor, in part by inhibiting glycolysis (Sebastián et al., 2012). Conditional deletion of *Sirt6* in intestinal epithelium in mice prone to developing intestinal polyps (*Apc^+/min^*) results in a threefold increase in adenoma number. These adenomas are larger, with a greater propensity for invasive behavior, than those forming in SIRT6-proficient mice. Treatment of these animals with dichloroacetate (DCA), which increases mitochondrial respiration mainly by inhibiting PDK1, suppresses tumorigenesis specifically in *Sirt6* conditionally-deleted mice (Sebastián et al., 2012). These findings highlight the importance of SIRT6-mediated suppression of glycolysis in tumor suppression.

With respect to SIRT1, an initial study revealed that SIRT1 activates HIF-2α, but not HIF-1α, by deacetylating three lysine residues in the C-terminus of HIF-2α (Dioum et al., 2009). Through this mechanism, SIRT1 was reported to increase the expression of the genes encoding erythropoietin, driving red blood cell synthesis; vascular endothelial growth factor A (*VEGFA*), stimulating angiogenesis; and manganese superoxide dismutase (MnSOD), lowering cellular levels of ROS (Dioum et al., 2009). In contrast, a number of subsequent studies showed that SIRT1 physically interacts with HIF-1α (Finley et al., 2011; Laemmle et al., 2012; Lim et al., 2010). SIRT1 regulates HIF1 activity by deacetylating the HIF-1α subunit. However, there are conflicting reports regarding how SIRT1-mediated deacetylation affects HIF-1. In one study, Lim and colleagues provided evidence that, at physiological oxygen tension, deacetylation of HIF-1α by SIRT1 blocks the recruitment of the p300 acetyltransferase to the promoters of HIF-1α target genes, and thus inhibits HIF-1α transcriptional activity. During hypoxia, decreased NAD^+^ levels inhibit SIRT1 activity (Zhang et al., 2007), resulting in high levels of acetylated and activated HIF-1α. Thus, Lim et al. concluded that SIRT1 negatively regulates HIF-1α-driven glycolysis (Lim et al., 2010). Conversely, Laemmle et al. found that SIRT1 positively regulates HIF1 transcriptional activity, and that a correlation exists between SIRT1 and HIF-1α protein (but not mRNA) levels (Laemmle et al., 2012). The latter findings were confirmed *in vivo* by treating mice bearing hepatocellular carcinoma xenografts with a SIRT1 inhibitor. SIRT1 inhibition reduced tumoral expression of HIF1 target genes such as *VEGFA*, resulting in tumors with low vascular density (Laemmle et al., 2012). Overall, although numerous studies have shown that SIRT1 deacetylates HIF-1α, the functional consequences of this deacetylation on HIF1-mediated metabolic reprogramming in cancer require further elucidation.

The mitochondrial sirtuin SIRT3 acts as a tumor suppressor protein by reducing intracellular ROS, which at elevated levels can contribute to carcinogenesis (Bell et al., 2011; Finley et al., 2011; Kim et al., 2010a; Zhang et al., 2013). One major pathway of cellular ROS detoxification occurs through MnSOD. SIRT3 deacetylates MnSOD to enhance its catalytic activity (Kim et al., 2010a; Qiu et al., 2010). SIRT3 also deacetylates and activates IDH2 to induce recycling of the antioxidant glutathione (Someya et al., 2010). The elevated ROS levels in SIRT3-deficient cells promote tumorigenesis through at least two distinct mechanisms. First, ROS induce nuclear genomic instability, and impair mitochondrial DNA integrity (Kim et al., 2010a; Singh, 2006). Second, elevated ROS levels can increase glycolysis by promoting HIF-1α stability (Bell et al., 2011; Finley et al., 2011). Under hypoxic conditions, increased ROS levels inhibit PHD activity, and thus stabilize HIF-1α and enhance HIF1 transcriptional output (Chandel et al., 1998; Gerald et al., 2004). Overexpression of SIRT3 or MnSOD, or treatment with the antioxidant N-acetylcysteine, reduces ROS levels and cellular proliferation in *SIRT3*-deficient mouse embryonic fibroblasts and in cancer cells (Bell et al., 2011; Kim et al., 2010a). Strikingly, *Sirt3* knockout mice spontaneously develop mammary cancer (Kim et al., 2010a). SIRT3 also suppresses Warburg-type metabolism by deacetylating the E1α subunit of PDC, as well as its associated kinase PDK1 and its phosphatase PDP1, leading to decreased E1α phosphorylation, increasing PDC activity and oxidative metabolism (Fan et al., 2014).

Consistent with a tumor suppressor function of SIRT3 in humans, *SIRT3* mRNA and protein levels are decreased in a large fraction of human breast cancers and other human malignancies, associated with deletion of the *SIRT3* gene locus (Finley et al., 2011; Kim et al., 2010a). However, it should be pointed out that recent work links an extra copy of the *SIRT3* gene with a tumor-prone phenotype in humans (Aury-Landas et al., 2013). Overall, current evidence suggests that, by maintaining low cellular ROS levels, SIRT3 inhibits glycolytic metabolism, and suppresses tumor development and progression.

Finally, the nuclear sirtuin SIRT7 has recently been reported to regulate HIF1. SIRT7 binds both HIF-1α and HIF-2α, reducing their stability and consequently their transcriptional activity. It is unknown how SIRT7 regulates HIF protein stability; this effect occurs independently of SIRT7 deacetylase activity, and SIRT7 does not regulate known HIF-1α degradation pathways (Hubbi et al., 2013). Whether SIRT7 plays a role in HIF-1α-mediated glycolysis during tumorigenesis remains to be elucidated. SIRT7 promotes malignant properties of tumor cells by deacetylating histone H3 at lysine 18 (Barber et al., 2012).

## Oncogenic MYC regulates biomass production in cancer

### The *MYC* oncogene

MYC, L-MYC (MYCL) and N-MYC (MYCN) are the three members of the oncogenic MYC transcription factor family. N-MYC and MYC possess overlapping functions, although expression of N-MYC is more restricted, being most abundant in developing brain and kidney, as well as in post-mitotic cells undergoing differentiation (Dang, 2012; Hirvonen et al., 1990; Malynn et al., 2000). MYC (c-MYC) heterodimerizes with its partner protein MAX (MYC-associated factor X) to bind specific DNA sequences, termed E-boxes, found in promoter regions of 30% of all genes (Dang et al., 2009). In this regard, recent data indicate that MYC functions as an amplifier of the expression of essentially all expressed genes, in a cell-type-specific manner (Lin et al., 2012; Nie et al., 2012). To regulate gene expression, the MYC-MAX complex requires interaction with other transcription factors such as E2F1 and HIF1. MYC positively regulates ribosomal biogenesis, glucose metabolism, and mitochondrial respiration in most cell types (Dang et al., 2009). Ribosomal genes are particularly important MYC targets in the context of cellular transformation; heterozygosity for a ribosomal gene (*L24*) is sufficient to attenuate MYC-driven oncogenesis in B-cells (Barna et al., 2008).

With respect to glucose metabolism and mitochondrial function, a complex interplay exists between MYC and the HIF proteins. Under hypoxic conditions, HIF1 inhibits MYC activity, either through interruption of MYC-MAX binding or through stimulation of proteasomal degradation of MYC (Corn et al., 2005; Gordan et al., 2007b). However, oxygen levels tend to fluctuate in tumors (Dewhirst, 2007). Therefore, in tumor cells, where MYC levels are generally elevated, HIF-1α will only inhibit MYC activity during short periods of severe hypoxia, whereas, at other times, the high MYC levels can drive cellular proliferation (Gordan et al., 2007b). In contrast to HIF1, HIF2 was reported to promote MYC-MAX heterodimerization under hypoxic conditions and enhance MYC activity (Gordan et al., 2007a). Because HIF2 is expressed in endothelial cells, it has been postulated that, via this mechanism, HIF2 stimulates endothelial proliferation and angiogenesis. However, endothelial-specific deletion of HIF-2α does not affect endothelial proliferation per se; instead, it results in defective tumor vessel formation and increased tumor hypoxia and apoptosis (Skuli et al., 2009).

Increased MYC activity is a feature of many diverse human tumors (Dang et al., 2008; Dang et al., 2009). A key function of MYC in cancers is regulating the absorption and metabolism of the non-essential amino acid glutamine. In 1955, Harry Eagle observed that cellular proliferation of normal and malignant cells in culture requires exogenous glutamine (Eagle, 1955). Glutamine uptake by cancer cells exceeds that of any other amino acid by tenfold, and glutamine deprivation of transformed cells induces apoptosis (Wise et al., 2008; Yuneva et al., 2007). The importance of glutamine in cancer cell survival and growth is due largely to its involvement in macromolecular synthesis (Dang, 2010; Wise and Thompson, 2010). Glutamine is essential for protein translation in cancer cells. When glutamine is imported into the cell via the amino acid transporter SLC1A5 (also known as ASCT2), a portion of it is exported out of the cell via the bidirectional amino acid transporter SLC7A5, concomitant with uptake of extracellular essential amino acids (EAAs). Intracellular EAAs activate mTORC1, a master regulator of protein translation (Wise and Thompson, 2010). In addition, glutamine and glucose are key nitrogen and carbon sources for synthesis of all non-essential amino acids except tyrosine (Wise and Thompson, 2010).

Glutamine can enter the TCA cycle via conversion to glutamate and subsequently to α-ketoglutarate (α-KG). A large fraction of α-KG is further converted to malate, pyruvate and subsequently lactate, which is secreted by the cell. As a by-product of the conversion of malate into pyruvate by malate dehydrogenase, NADP^+^ is converted into NADPH. Hence, glutamine metabolism generates a substantial fraction of the NADPH that is essential for nucleotide and lipid synthesis, and cellular proliferation (DeBerardinis et al., 2007; Vander Heiden et al., 2009). Furthermore, the cancer cell generates mitochondrial oxaloacetic acid (OAA) from glutamine metabolism (DeBerardinis et al., 2007). OAA and acetyl-CoA condense to form citrate, which is exported from mitochondria into the cytosol. Here, citrate can liberate acetyl-CoA for lipid synthesis, whereas the remaining OAA is converted to pyruvate (Wise and Thompson, 2010). Through these processes, glutamine acts as an important substrate for energy production (DeBerardinis et al., 2007), as a carbon and nitrogen source to support protein, nucleotide and lipid synthesis (Jones and Thompson, 2009; Wise and Thompson, 2010), and as a means to generate NADPH (Wise and Thompson, 2010). Recent studies have revealed that, in cancer cells with impaired mitochondrial function, or under hypoxic or pseudo-hypoxic conditions, glutamine is converted to α-KG and then to citrate via reductive carboxylation, which is subsequently employed as a precursor for lipid synthesis and to replenish TCA cycle intermediates (Metallo et al., 2012; Mullen et al., 2012).

To summarize, MYC regulates glutamine uptake and metabolism in cancer cells in at least three distinct ways. First, MYC directly increases the expression of the amino acid transporters SLC5A1 and SLC7A1 (Gao et al., 2009). Second, MYC stimulates the conversion of glutamine to glutamate by indirectly regulating the expression of the enzyme glutaminase (GLS). MYC transcriptionally represses miR-23a and miR-23b, microRNAs that bind the 3′ untranslated region (UTR) of *GLS* mRNA and induce its degradation (Gao et al., 2009). Finally, MYC promotes the expression of several enzymes involved in nucleotide biosynthesis using glutamine (Dang, 2010; Wise and Thompson, 2010).

### Interplay between MYC and sirtuins

Multiple sirtuins (SIRT1, SIRT2, SIRT6 and SIRT7) directly modulate MYC function (Fig. 3B and Table 1). SIRT6 regulates MYC activity similarly to HIF1; it deacetylates acH3K56 residues at MYC target genes, inhibiting MYC transcriptional output. A large fraction of MYC target gene promoters are enriched for SIRT6; however, SIRT6 does not regulate MYC-mediated glucose uptake or glycolytic gene expression. Instead, SIRT6 specifically suppresses expression of MYC genes involved in ribosomal biogenesis (e.g. *Rpl3*, *Rpl6* and *Rpl23*) and glutamine metabolism (*Gls*) (Sebastián et al., 2012). This specificity implies that additional cofactor(s) are likely involved in SIRT6 regulation of MYC-mediated ribosomal biogenesis. Intriguingly, although these MYC target genes are upregulated in SIRT6-deficient tumors, their expression is not altered in *Sirt6* knockout mouse embryonic fibroblasts. This suggests that the upregulation of ribosomal biogenesis genes in the absence of SIRT6 occurs as a fairly late event in tumor development (Sebastián et al., 2012). Decreased SIRT6 protein levels and deletion of the *SIRT6* gene occur in human colon, pancreatic and hepatocellular carcinomas, and in cancer cell lines, consistent with a tumor suppressor role for human SIRT6 (Lin et al., 2013; Sebastián et al., 2012; Zhang and Qin, 2013). SIRT6 itself is also subject to post-translational regulation in cancers. USP10 is a deubiquitylase that normally enhances SIRT6 protein stability. Reduced SIRT6 protein levels and consequently increased MYC activity can result from reduced USP10 levels in colon tumors (Lin et al., 2013).

In contrast to SIRT6, SIRT1 cooperates with MYC family proteins. Increased MYC activity provides cancer cells with a growth advantage, in part by inhibiting activity of the p53 tumor suppressor through SIRT1 (Menssen et al., 2012). MYC drives SIRT1 protein expression. Consequently, higher SIRT1 activity results in increased deacetylation of p53, reducing p53 activity (Menssen et al., 2012). Multiple studies have demonstrated a positive correlation between MYC and SIRT1 expression in various human cancers, including hepatocellular carcinoma (Jang et al., 2012) and colorectal cancer (Kriegl et al., 2012; Menssen et al., 2012). Elevated expression of MYC and SIRT1 is associated with higher grades of malignancy (Jang et al., 2012; Kriegl et al., 2012). Inhibiting SIRT1 with siRNA or the sirtuin inhibitor nicotinamide induces cellular senescence and apoptosis in cells with dysregulated MYC expression (Jang et al., 2012; Menssen et al., 2012). Despite conflicting reports, it is generally accepted that MYC can bind to the *SIRT1* promoter to induce its expression (Mao et al., 2011; Yuan et al., 2009), and SIRT1 in turn can deacetylate the C-terminus of MYC (Mao et al., 2011; Menssen et al., 2012; Yuan et al., 2009). Initially, Yuan and colleagues found that deacetylated MYC is prone to degradation, suggesting that SIRT1 reduces MYC protein levels and inhibits its function (Yuan et al., 2009). However, more recent studies have provided evidence that MYC deacetylation results in its stabilization (Menssen et al., 2012) and enhances MYC-MAX association and activity (Mao et al., 2011). Menssen et al. proposed a model in which MYC stabilizes SIRT1 protein levels and enhances its activity indirectly, by increasing levels of nicotinamide-phosphoribosyltransferase (NAMPT), which catalyzes the first step in NAD^+^ synthesis, and by binding and sequestering the SIRT1 inhibitor DBC1 (Menssen et al., 2012).

In addition to MYC, SIRT1 also forms a positive feedback loop with N-MYC, whereby N-MYC enhances *SIRT1* expression and SIRT1 inhibits N-MYC proteasomal degradation by promoting N-MYC phosphorylation (through MKP3, which dephosphorylates and inactivates ERK) (Marshall et al., 2011). It is currently unknown whether SIRT1 regulates the metabolic effects of MYC in cancer; however, based on these findings, it is likely that SIRT1 can induce expression of MYC target genes important in mediating metabolic reprogramming. Indeed, two reports demonstrated that SIRT1 stimulates MYC-induced *LDHA* expression (Mao et al., 2011; Vettraino et al., 2013).

The MYC oncoproteins also form a positive feedback loop with SIRT2. MYC and N-MYC upregulate *SIRT2* expression, and SIRT2 inhibits the ubiquitylation and degradation of both of these MYC proteins by suppressing expression of NEDD4, an E3 ubiquitin-protein ligase (Liu et al., 2013a). By promoting MYC protein stabilization, SIRT2 can enhance growth of neuroblastoma and pancreatic cancer cells (Liu et al., 2013a). Importantly, however, other studies point to roles for SIRT2 in tumor suppression via maintenance of genomic stability (Kim et al., 2011).

Recently, SIRT7 was identified as another suppressor of MYC function. In response to endoplasmic reticulum (ER) stress, XBP1, a regulator of the unfolded protein response (UPR), binds to the *SIRT7* promoter to induce transcription. SIRT7 in turn is recruited by MYC to block MYC-mediated transcription of ribosomal genes, to inhibit cellular protein translation. Suppression of ribosomal biogenesis by SIRT7 requires its catalytic activity, suggesting that SIRT7 reduces MYC activity by acH3K18 deacetylation (Barber et al., 2012). In this context, one report indicates that SIRT7 protects against fatty liver formation in mice (Shin et al., 2013). This interplay between SIRT7 and MYC might suggest that SIRT7 functions as a tumor suppressor by inhibiting MYC activity, similar to SIRT6. However, a previous study demonstrated that hypoacetylation of H3K18 is associated with tumorigenesis and poor clinical outcome, and that SIRT7 is essential in maintaining low levels of acH3K18 in cancer cells (Barber et al., 2012). Further studies are needed to test whether SIRT7 might function as a tumor suppressor in some contexts.

The mitochondrial sirtuins have not been definitively linked to MYC protein family function. SIRT5 has been reported to interact weakly with MYC (Sebastián et al., 2012), a finding currently of uncertain biological significance. More generally, SIRT5 is a candidate to promote metabolic reprogramming in cancer; it regulates metabolic complexes implicated in neoplasia, and suppresses mitochondrial respiration (Park et al., 2013). No physical interplay between MYC and SIRT3 or SIRT4 has been reported. However, SIRT4, like MYC, does play a major role in regulating glutamine metabolism. SIRT4 overexpression can prevent MYC-induced glutamine dependency and tumor growth (Jeong et al., 2014). As previously noted, glutamine is converted to α-KG in the mitochondria. This conversion requires glutamate dehydrogenase (GDH), an enzyme that is ADP-ribosylated and inactivated by SIRT4. Thus, SIRT4 inhibits mitochondrial glutamine metabolism (Haigis et al., 2006), suggesting that it could act as a tumor suppressor. Consistent with this hypothesis, SIRT4-deficient mice spontaneously develop a wide spectrum of tumors, and the absence of SIRT4 can accelerate cellular growth in MYC-induced B-cell lymphomas (Jeong et al., 2014; Jeong et al., 2013). In response to DNA damage, SIRT4 inhibits glutamine metabolism to promote cell cycle arrest, allowing for DNA damage repair (Csibi et al., 2013; Jeong et al., 2014; Jeong et al., 2013). mTORC1 (mammalian target of rapamycin complex 1), which regulates protein synthesis and whose kinase activity is upregulated in many human cancers, alleviates SIRT4-induced inhibition of glutamine metabolism by suppressing SIRT4 expression (Csibi et al., 2013). Conversely, SIRT4 overexpression inhibits glutamine metabolism in Burkitt lymphomas with high MYC activity, reduces their proliferation, and sensitizes them to glucose depletion (Jeong et al., 2014). Finally, multiple human tumor types show reduced SIRT4 levels (Csibi et al., 2013; Jeong et al., 2014). Altogether, these studies provide compelling evidence that SIRT4 activation could be a rational therapeutic strategy in treating tumors with elevated MYC activity and glutamine dependency (Jeong et al., 2014).

## Concluding remarks

Overall, studies have shown that there is a multi-faceted interplay between mammalian sirtuin proteins and the MYC and HIF transcription factor families (Table 1 and Fig. 3), potentially rendering some of the sirtuins attractive therapeutic targets to reverse metabolic reprogramming in cancer cells. Whereas the preponderance of the evidence suggests that SIRT1 in many contexts promotes cancer metabolism by working in conjunction with HIF and MYC family proteins, SIRT3, SIRT4 and SIRT6 all inhibit distinct aspects of the metabolic alterations observed in tumor cells. SIRT3 inhibits HIF activity by maintaining low cellular ROS levels; SIRT4 blocks MYC-mediated glutamine metabolism by inhibiting GDH; and SIRT6 attenuates transcriptional activity of HIF1 and MYC via effects on chromatin.

Cellular transformation generally requires both inactivation of tumor suppressor genes and increased activity of proto-oncogenes (Sherr, 2004). The absence of SIRT6 in immortalized mouse embryonic fibroblasts is sufficient to confer upon them tumorigenic potential (Sebastián et al., 2012). Thus, in this cellular context, SIRT6, by regulating HIF1 and/or MYC, qualifies as a tumor suppressor. Activation of SIRT6 in preformed cancers might conceivably represent a strategy to inhibit signaling through these oncogenic transcription factors. However, it is currently unknown whether elevated SIRT6 activity confers increased tumor suppression capacity. One report found that overexpression of SIRT6 induces apoptosis in diverse cancer cell types, but not in normal cells, although this effect was dependent on the mono-ADP-ribosyltransferase activity of SIRT6, likely implying that SIRT6-induced apoptosis represents a function of SIRT6 distinct from its metabolic roles (Van Meter et al., 2011). Similarly, SIRT6 overexpression *in vivo* seems to provide some protection against lung cancers in mice (Kanfi et al., 2012; Lombard and Miller, 2012).

Although we have focused on functions of SIRT6 in regulating MYC and HIF, it is very likely that other roles of SIRT6 are also relevant for its tumor suppressor capacity. As previously noted, SIRT6 promotes maintenance of genomic stability via multiple mechanisms. In addition, SIRT6 represses the activities of the lipogenic transcription factors SREBP1 and SREBP2 (Elhanati et al., 2013; Tao et al., 2013). Because an uninterrupted supply of lipids is crucial for tumor growth, maintenance of SREBP activity is likely important for the rapid proliferation of cancer cells, a hypothesis that has been confirmed in the context of a subset of gliomas (Guo et al., 2011). The role of SIRT6 in co-repressing JUN and consequently suppressing IIS is also likely relevant in this context (Sundaresan et al., 2012). JUN is itself a proto-oncogene, and increased IIS occurs in many diverse tumor types (Wong et al., 2010).

However, other data point to a Janus-faced role of SIRT6 in neoplasia. Elevated SIRT6 levels have been reported in pancreatic (Bauer et al., 2012), breast (Khongkow et al., 2013) and prostate (Liu et al., 2013b) carcinomas where SIRT6 contributes to cell migration, enhanced cell viability and chemotherapeutic resistance. Thus, SIRT6 can assume an oncogenic role in some contexts. In the setting of normal cells, SIRT6 might provide protection against transformation by suppressing metabolic reprogramming and maintaining genomic integrity. However, in some tumor types, SIRT6 function might be recruited to promote stress resistance, i.e. against genomic insult or other forms of cellular injury (Jedrusik-Bode et al., 2013; Miteva and Cristea, 2014; Simeoni et al., 2013). Given the pleiotropic nature of SIRT6’s roles in the cell, it is likely that additional major functions of this protein exist in normal and transformed cells that remain to be identified. Clearly, further studies are needed to clarify the opposing roles of SIRT6 in cancer, and to investigate the therapeutic potential of SIRT6 in established tumors.
